# The Influence of Aortic Pulse Wave Velocity on Short-Term Functional Capacity in Patients with Mild Paravalvular Regurgitation Following Transcatheter Aortic Valve Implantation

**DOI:** 10.21470/1678-9741-2019-0454

**Published:** 2020

**Authors:** Ibrahim Halil Kurt, Ömer Şen, Mehmet Kuçükosmanoğlu, Fatma Özge Salkın, Örsan Deniz Urgun, Şeyda Şahin, Salih Çolak, Salih Kılıç

**Affiliations:** 1 Department of Cardiology, Health Sciences University, Adana Research and Training Hospital, Adana, Turkey.

**Keywords:** Transcatheter Aortic Valve Replacement, Aortic Valve Insufficiency, Pulse Wave Analysis, Blood Pressure, Reference Values, Aortic Valve Stenosis, Regression Analysis

## Abstract

**Introduction:**

Recently, the clinical significance of mild paravalvular aortic regurgitation (PAR) has been evaluated and suggested that it can be predictor of clinical outcomes. In our study, we aimed to investigate the interaction of aortic pulse wave velocity (PWV) and mild PAR and their effects on the functional status of patients after transcatheter aortic valve implantation (TAVI).

**Methods:**

A total of 109 consecutive patients with symptomatic severe aortic stenosis were enrolled prospectively. After TAVI procedure, they were divided in to three groups according to PAR and PWV measurements. Patients without PAR were defined as the NonePAR group (n=60), patients with mild PAR and normal PWV were defined as the MildPAR-nPWV group (n=23), and patients with mild PAR and high PWV were defined as the MildPAR-hPWV group (n=26).

**Results:**

Compared with other groups, the MildPAR-hPWV group was older (*P*<0.001), hypertensive (*P*=0.015), and had a higher pulse pressure (*P*=0.018). In addition to PWV, this group had lower aortic regurgitation index (ARI) (*P*=0.010) and higher rate of New York Heart Association (NYHA) class II (at least) patients (*P*<0.001) in 30-day follow-up period. On multivariate regression analysis, the MildPARhPWV group (odds ratio=1.364, 95% confidence interval 1.221-1.843; *P*=0.011) as well as N-terminal-pro-brain natriuretic peptide levels and ARI were independently related with 30-day functional NYHA classification. However, NonePAR or MildPAR-nPWV group was not an independent predictor of early functional status.

**Conclusion:**

It was concluded that high PWV may adversely affect early functional status in patients with mild PAR in contrast to normal values following TAVI.

**Table:** 

Abbreviations, acronyms & symbols				
ACE-I/ARB	= Angiotensin converting enzyme inhibitor/angiotensin II receptor blocker		LVMI	= Left ventricular mass index
AF	= Atrial fibrillation		MAP	= Mean arterial pressure
ANOVA	= Analysis of variance		MildPAR-hPWV	= Mild paravalvular aortic regurgitation with high pulse wave velocity
AR	= Aortic regurgitation			
ARI	= Aortic regurgitation index		MildPAR-nPWV	= Mild paravalvular aortic regurgitation with
AS	= Aortic stenosis		NonePAR	= No paravalvular aortic regurgitation
AVA	= Aortic valve area		NT-proBNP	= N-terminal pro-brain natriuretic peptide
AVMG	= Aortic valve mean gradient		NYHA	= New York Heart Association
AVPG	= Aortic valve peak gradient		AR	= Paravalvular aortic regurgitation
BMI	= Body mass index		PP	= Pulse pressure
CABG	= Coronary artery bypass grafting		PSAX	= Parasternal short axis
cDia	= Central diastolic pressure		PWA	= Pulse wave analysis
CI	= Confidence interval		PWV	= Pulse wave velocity
cPP	= Central pulse pressure		SBP	= Systolic blood pressure
cSys	= Central systolic pressure		STS	= Society of Thoracic Surgeons
DBP	= Diastolic blood pressure		TAVI	= Transcatheter aortic valve implantation
DM	= Diabetes mellitus		TEE	= Transesophageal echocardiography
HDL-C	= High-density lipoprotein cholesterol		TTE	= Transthoracic echocardiography
LDL-C	= Low-density lipoprotein cholesterol		VARC	= Valve Academic Research Consortium
LVEF	= Left ventricular ejection fraction		WBC	= White blood cell

## INTRODUCTION

Aortic stenosis (AS) is a common valve disease in elderly people and its prevalence increases 10% after 80 years old^[1]^. It is a degenerative and an atherosclerotic-like process that involves both vessels and aortic valve^[2,3]^. Pathologically increased calcium and collagen ratio leads to arterial stiffness and AS^[4]^. When the aorta becomes stiffer, the elastic capacity decreases and aortic pulse wave velocity (PWV) increases. It is known that elastic recoil of the aorta maintains the perfusion pressure of the tissues during diastole after aortic valve closure. The velocity of pressure wave is affected by elastic properties of comprised vessel. Increased aortic stiffness leads to increase in PWV and cardiac afterload^[5,6]^. The correlation of PWV and cardiovascular disease also has been well established^[7-9]^.

On the other hand, transcatheter aortic valve implantation (TAVI) has become the main therapeutic approach for surgical high-risk or inoperable patients with symptomatic severe AS^[10,11]^. In literature, sutureless implantation and incomplete circumferential apposition of the valve at aortic annulus are regarded as the main causes of paravalvular aortic regurgitation (PAR). It is known that mild PAR after TAVI procedure has no adverse effects on cardiovascular outcomes^[12,13]^. However, in some recent studies, the clinical significance of mild PAR has been evaluated and suggested that it can be predictor of clinical outcomes^[13,14]^. In our study, we aimed to investigate the interaction of aortic PWV and mild PAR and their effects on the functional status of patients after TAVI procedure.

## METHODS

### Patient Population

Between May 2016 and June 2018, 188 consecutive patients with symptomatic severe AS were evaluated by a heart team, and 129 of them were enrolled in the study prospectively. Among these, 20 patients were excluded from the study due to comorbidities that have significant effect on PWV measurement or functional status (five patients had more than mild aortic regurgitation [AR], five patients had left ventricular ejection fraction [LVEF] < 45%, one patient had disabling stroke, four patients had chronic kidney disease, three patients had thoracic or abdominal aneurysm, and two patients had severe pulmonary disease), remaining 109 patients. The Ethics Committee approved the study protocol, and informed consent was obtained from all patients.

The patients were evaluated one day before the TAVI procedure and at the first month after it. Before TAVI, full clinical and medical history, physical examination including height and weight, and routine blood samples including N-terminal pro-brain natriuretic peptide (NT-proBNP) were collected. In the Reference Values for Arterial Stiffness Collaboration study published in 2010, patients were divided into normal and high PWV groups^[7]^. In this study, after TAVI procedure, patients were divided into three groups according to their PAR and PWV measurements. Patients without PAR were defined as the NonePAR group (n=60), patients with mild PAR and normal PWV were defined as the MildPAR-nPWV group (n=23), and patients with mild PAR and high PWV were defined as the MildPAR-hPWV group (n=26).

### Pulse Wave Velocity

**Fig. 1:**
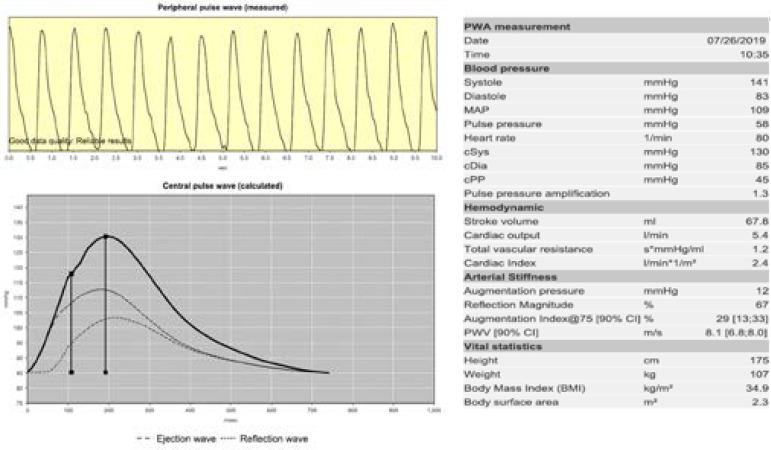
Analysis of a patient. cDia=central diastolic pressure; CI=confidence interval; cPP=central pulse pressure; cSys=central systolic pressure; MAP=mean arterial pressure; PWA=pulse wave analysis; PWV=pulse wave velocity

Measurements were made according to previously determined international AS measurement methods and recommendations^[15]^. In patients eligible for TAVI, all medications that would affect the vascular tone, caffeine, and alcohol were withheld at least 24 hours before the measurement. To prevent inter-measurement variability, a single observer, unaware of patient data, received all measurements. At least three consecutive measurements were performed. Arterial stiffness was measured from the right brachial region after resting for at least 15 minutes, in a silent room, at appropriate room temperature (22-25 ˚C) to minimize the artefacts, in complete basal resting condition, in supine position, and using a Mobil-O-Graph^®^ ARC solver algorithm (IEM GmbH, Stolberg, Germany). This measurement algorithm gives us simultaneous measurement of brachial blood pressure with approved assessment of AS parameters, such as PWV and Augmentation Index^[16,17]^. PWV calculation includes dynamically measured results and individual-related values. Aortic pressure, stroke volume, flow, and pressure curves are evaluated simultaneously to establish the association with individual PWV (Figure 1). Before and after the first month of procedure, PWV values were assessed with ARC solver algorithm. According to the Reference Values for Arterial Stiffness Collaboration, we used 10.5 m/sn as the cut-off value for PWV in our study^[7]^. However, patients who were older than 70 years old, had 10.6 m/sn median PWV value in their data.

### Echocardiography

All patients underwent transthoracic echocardiography (TTE) before, early after, and one month after TAVI procedure. We used TTE instead of transesophageal echocardiography (TEE) during the procedure for PAR grading because all procedures were performed under minimal sedation and local anaesthesia. Pre and post-procedural valve function were evaluated according to the Valve Academic Research Consortium (VARC)-2 and the European Association of Echocardiography/American Society of Echocardiography guidelines by TTE^[18,19]^. Standard two-dimensional, M-mode, and Doppler echocardiographic evaluation were performed by two experienced echocardiographers using a dedicated ultrasound machine (ACUSON SC2000 PRIME Ultrasound System, Siemens Medical Solutions, United States of America) with a 2.5-3.5 and Z6 MHz transducer. Echocardiographers were blinded to patients’ clinical and laboratory data and evaluated patients independently. Post-TAVI PAR degree was evaluated 10 minutes after valve implantation or final post-dilatation. Parasternal long axis and parasternal short axis (PSAX) views with colour Doppler imaging were used to determine PAR. In PSAX, the area of the circumferential aortic jet was used to grading the clinical significance of PAR (< 10%: mild, 10% to 29%: moderate, and 30%: severe PAR)^[19]^. LVEF was obtained by the Simpson’s method.

### Procedure and Hemodynamic Assessment

We performed transfemoral TAVI for all patients with minimal sedation with midazolam and local anaesthesia. The femoral artery was accessed and closed percutaneously by using two Perclose ProGlide^TM^ systems (Abbott Vascular Devices, Redwood City, California, United States of America). After serial dilatation of access site, we advanced the appropriate sheath over the stiff wire.

After heparin injection for maintaining effective anticoagulation, temporary pacemaker lead and pigtail catheter were placed. We measured aortic and left ventricular, systolic, and diastolic pressures before valve implantation. We used self-expandable bioprosthetic valves (CoreValve^TM^ [Medronic Inc; Minneapolis, Minnesota, United States of America] or Portico^TM^ [St. Jude Medical, Minneapolis, Minnesota, United States of America]) for all patients.

At least 10 minutes after the valve implantation or postdilation, we measured pressures again within the heart rate between 60-80 beat/min. We also used aortic regurgitation index (ARI) for objective and quantitative assessment of PAR during TAVI procedure. ARI is a ratio of difference between diastolic pressures of aorta and left ventricular to systolic pressure of aorta. It has been validated before and has an inverse correlation with PAR after TAVI. In addition, it has shown that cut-off value of ARI 25 had 95%-100% negative predictive value for more than mild PAR^[13,20]^.

### Statistical Analysis

Statistical analysis was performed using SPSS Inc. Released 2008, SPSS Statistics for Windows, Version 17.0, Chicago: SPSS Inc (United States of America). Baseline, clinical, hemodynamic, echocardiographic, and laboratory parameters of study patients were summarized as percentages and frequencies for categorical variables and mean (± standard deviation) for continuous variables. Continuous variables were analyzed by the analysis of variance test. Chi-square test was used for comparing the categorical variables. Univariate analysis was used for obtaining the effects of different variables on New York Heart Association (NYHA) functional class.

The variables with an unadjusted *P*-value < 0.20 in bivariate analysis were entered in the multivariate logistic regression analysis. All significant parameters in the univariate analysis were selected in the multivariate model. Multivariate, stepwise backward conditional logistic regression analysis was used to obtain the independent predictors of NYHA functional status. Two-tailed *P*-value < 0.05 was considered as statistically significant.

## RESULTS

The mean age of the study group was 77.6±5.1 years; 62 (55.2%) patients were female, and 47 (44.8%) were male. MildPAR-hPWV group was older and the number of patients with hypertension in this group was higher than in other groups (Table 1). The other baseline characteristics, including the Society of Thoracic Surgeons score (*P*=0.618), were similar within groups (Table 1).

**Table 1:** Patients' baseline characteristics.

Variables	NonePAR group (n=60)	MildPAR-nPWV group (n=23)	MildPAR-hPWV group (n=26)	*P*-value (ANOVA)
Age (years)	75.5±4.5^a^	76.8±4.5^b^	81.1±5,1	< 0.001
Sex (male), n (%)*	23 (38,3)	11 (48.8)	13 (50)	0.966
BMI (kg/m^2^)	27±5,6	27±4.9	26±6.2	0.904
Hypertension, n (%)*	25 (41.7)^c^	7 (30.4)^d^	18 (69.2)	0.015
DM, n (%)*	15 (25)	6 (26.1)	8 (30.8)	0.417
Smoking, n (%)*	10 (16.7)	5 (21.7)	7 (26.9)	0.548
Previous CABG, n (%)*	14 (23.3)	6 (26.1)	8 (30.8)	0.859
Coronary artery disease, n (%)*	32 (53.3)	10 (43.5)	11 (42.3)	0.341
AF, n (%)*	12 (20)	6 (23)	6 (26)	0.234
STS score (%)	11.2±2.7	11.8±2.9	11.15±2.1	0.618
Medication, n (%)*				
Aspirin	43 (71.7)	18 (69.2)	17 (74)	0.623
Statin	16 (26.6)	8 (31)	7 (30)	0.186
β-blocker	28 (46.6)	14 (54)	12 (52.1)	0.324
ACE-I/ARB	16 (26.6)	9 (34.6)	7 (30)	0.254
WBC count ('1000/pl)	13.1±3.98	12.8±4.78	13.2±3.72	0.266
Hemoglobin (mg/dl)	11.1±1.62	10.9±1.85	11±1.36	0.522
Platelet count ('109/l)	188±68	176±52	196±44	0.346
Creatinine (mg/dl)	0.91±0.24	0.86±0.20	0.88±0.36	0.286
Total cholesterol (mg/dl)	192.2±28.6	186.8±33	189.3±25	0.428
HDL-C (mg/dl)	39.6±8.4	40.2±9.9	38.7±7.8	0.312
LDL-C (mg/dl)	127±35.5	132±29	125±41	0.218
Triglyceride (mg/dl)	135±65	125±86	140±115	0.313

X^2^

a *P*<0,001 vs. MildPAR-hPWV group;

b *P*<0.001 vs. MildPAR-hPWV group;

c *P*=0.052;

d *P*=0.019 vs. MildPAR-hPWV group

Significant *P*-values (P<0.05) are indicated in boldface ACE-I/ARB=angiotensin converting enzyme inhibitor/angiotensin II receptor blocker; AF=atrial fibrillation; ANOVA=analysis of variance; BMI=body mass index; CABG=coronary artery bypass grafting; DM=diabetes mellitus; HDL-C=high-density lipoprotein cholesterol; LDL-C=low-density lipoprotein cholesterol; MildPAR-hPWV=mild paravalvular aortic regurgitation with high pulse wave velocity; MildPAR-nPWV=mild paravalvular aortic regurgitation with normal pulse wave velocity; NonePAR=no paravalvular aortic regurgitation; STS=Society of Thoracic Surgeons; WBC=white blood cell

**Fig. 2:**
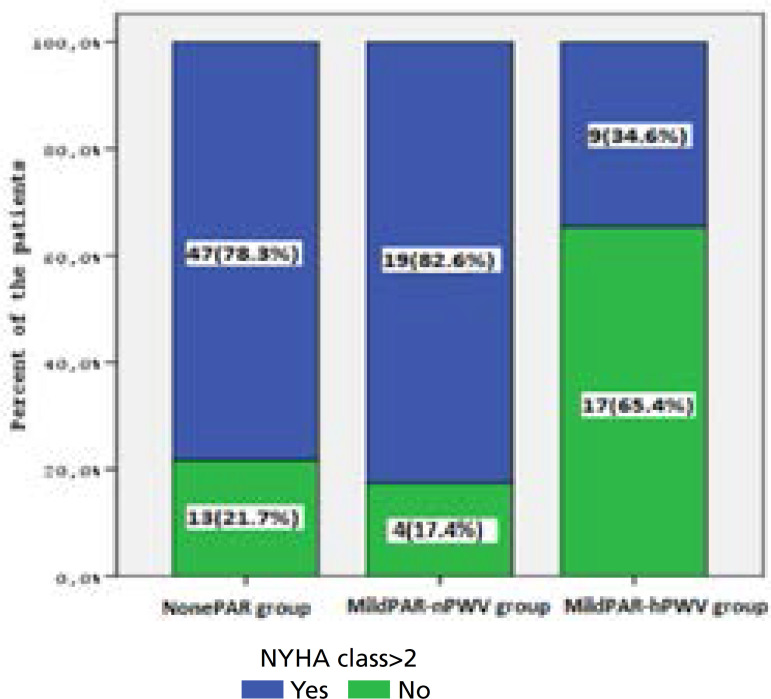
Distribution of NYHA classification within groups. MildPARhPWV= mild paravalvular aortic regurgitation with high pulse wave velocity; MildPAR-nPWV=mild paravalvular aortic regurgitation with normal pulse wave velocity; NonePAR=no paravalvular aortic regurgitation; NYHA=New York Heart Association

**Fig. 3:**
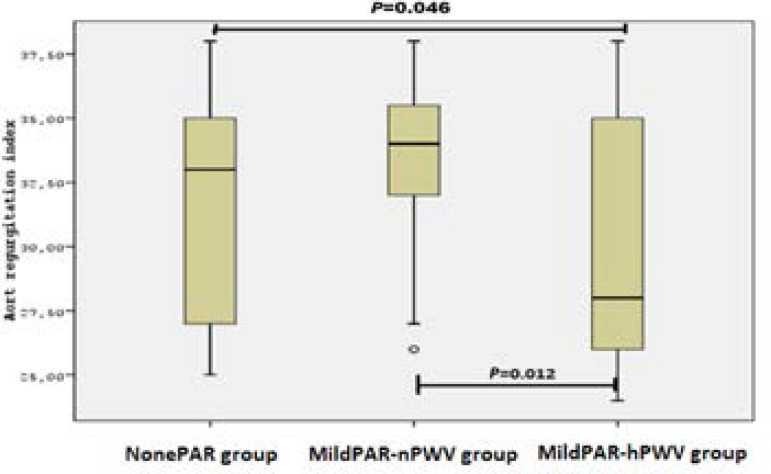
Aortic regurgitation index within groups. MildPAR-hPWV=mild paravalvular aortic regurgitation with high pulse wave velocity; MildPAR-nPWV=mild paravalvular aortic regurgitation with normal pulse wave velocity; NonePAR=no paravalvular aortic regurgitation

The patients’ pre-and post-TAVI echocardiographic measurements including aortic valve area, aortic gradients, more than mild mitral and AR degree, left ventricular mass index, and LVEF were found similar within groups. Pre-TAVI, MildPAR-hPWV group had significant pulse pressure (PP) value (NonePAR 51.5±12.9 *vs*. MildPAR-nPWV 50.8±12.6 *vs*. MildPAR-hPWV 60.2±8.9; *P*=0.018), in contrast to post-TAVI values (*P*=0.067). MildPAR-hPWV group also had lower ARI than other groups (NonePAR 31.8±4.1 *vs*. MildPAR-nPWV 32.9±3.5 *vs*. MildPAR-hPWV 29.3±4.7; *P*=0.010). Distributions of ARI within groups are shown in Figure 2. Totally, 22 (20.2%) patients needed permanent pacemaker implantation due to heart block after TAVI, however there was no significant difference within groups (*P*=0.654). In addition, MildPAR-hPWV group also had a higher rate of NYHA class II (at least) patients (NonePAR 21.7% *vs*. MildPAR-nPWV 17.4% *vs*. MildPAR-hPWV 64.4%; *P*<0.001) in a 30-day follow-up period. Distribution of NYHA class > II within groups is shown in Figure 3. First-month NT-proBNP levels (*P*=0.009) were also found higher in the MildPAR-hPWV group than in the other groups (Table 2).

**Table 2:** Patients' clinic, hemodynamic, and echocardiographic characteristics.

Variables	NonePAR group (n=60)	MildPAR-nPWV group (n=23)	MildPAR-hPWV group (n=26)	*P*-value (ANOVA)
Pre-TAVI SBP (mmHg)	121±16	119±14	123.1±13.4	0.765
Post-TAVI SBP (mmHg)	119±18	122±21	121±24	0.767
Pre-TAVI DBP (mmHg)	67.5±8.3	67±6.8	64.3±8.9	0.333
Post-TAVI DBP (mmHg)	68.4±8.2	68.2±8.6	66±8.2	0.346
Pre-TAVI MAP (mmHg)	91±9.2	88.7±8.2	85.6±10.4	0.112
Post-TAVI MAP (mmHg)	91.7±12	92.2±13	86.9±11	0.230
Pre-TAVI PP (mmHg)	51.5±12.9^a^	50.8±12.6^b^	60.2±8.9	0.018
Post-TAVI PP (mmHg)	50.8±12.7	54.8±15.5	59.6±15.6	0.067
Pre-TAVI pulse (beat/min)	72±13	68±9	68±15	0.369
Post-TAVI pulse (beat/min)	74±16	69±12	76±11	0.286
Pre-TAVI AVA (cm^2^)	0.75±0.9	0.74±0.07	0.76±0.11	0.891
Post-TAVI AVA (cm^2^)	2.08±0.27	2.11±0.18	2.14±1.9	0.842
Pre-TAVI AVPG (mmHg)	77±11	76±10	79±10.5	0.480
Post-TAVI AVPG (mmHg)	11±4	14±3	10±4	0.384
Pre-TAVI AVMG (mmHg)	47.1±6.2	46.5±5.5	48.5±7	0.344
Post-TAVI AVMG (mmHg)	5±3	7±4	6±3	0.524
LVEF(%)	55±10.4	54±9.6	55±7.6	0.756
LVMI (g/m^2^)	122.6±26	121.8±31	123±24	0.652
Post-TAVI NYHA class, n(%)	13 (21.7)	4 (17.4)	17 (64.4)	< 0.001
Permanent pacemaker implantation, n (%)	11 (19)	5 (21.7)	6 (23.1)	0.654
Pre-TAVI AR > Mild, n (%)	7 (11.6)	4 (15.3)	3 (13)	0.284
AR index	31.8±4.1^c^	32.9±3.5^d^	29.3±4.7	0.010
Pre-TAVI PWV (m/sec)	10.2±1.7	9.7±1.8	12.2±1.2	< 0.001
Post-TAVI PWV (m/sec)	10±1.7	9.6±1.9	12.3±1.4	< 0.001
Nt-ProBNP (pg/ml)	613±448^e^	572±414^f^	913±438	0.009

Significant *P*-values (*P*<0.05) are indicated in boldface.

a *P*=0,070 vs. MildPAR-hPWV group;

b *P*=0.023 vs. MildPAR-hPWV group

c *P*=0.046 vs. MildPAR-hPWV group;

d *P*=0.012 vs. MildPAR-hPWV group

e *P*=0.015 vs. MildPAR-hPWV group;

f P =0.026 vs. MildPAR-hPWV group

ANOVA=analysis of variance; AR=aortic regurgitation; AVA=aortic valve area; AVMG=aortic valve mean gradient; AVPG=aortic valve peak gradient; DBP=diastolic blood pressure; LVEF=left ventricular ejection fraction; LVMI=left ventricular mass index; MAP=mean arterial pressure; MildPAR-hPWV=mild paravalvular aortic regurgitation with high pulse wave velocity; MildPAR-nPWV=mild paravalvular aortic regurgitation with normal pulse wave velocity; NonePAR=no paravalvular aortic regurgitation; NT-proBNP=N-terminal pro-brain natriuretic peptide; NYHA=New York Heart Association; PP=pulse pressure; PWV=pulse wave velocity; SBP=systolic blood pressure; TAVI=transcatheter aortic valve implantation

On multivariate regression analysis, the MildAR-hPWV group (odds ratio=1.364, 95% confidence interval 1.221-1.843; *P*=0.011) as well as NT-proBNP levels and ARI were independently associated with 30-day functional NYHA classification. However, NonePAR or MildPAR-nPWV group was not an independent predictor of early functional status.

## DISCUSSION

In our study, we have shown that patients who had high PWV together with mild PAR following TAVI procedure had worse early functional status as compared with none PAR patients or mild PAR patients with normal PWV. To the best of our knowledge, this is the first study to investigate the clinical significance of mild PAR subgroups after TAVI procedure. We also demonstrated that the NT-proBNP levels and hemodynamic ARI were independently associated with the early functional status in these patients.

There are various cardiovascular risk factors such as age, hypertension, diabetes mellitus, and chronic kidney disease contributing to aortic stiffness^[7-9]^. It is also known that aortic stiffness is a good predictor of concomitant cardiovascular diseases and can be measured by non-invasive PWV measurements^[8,16,17]^. Accordingly, MildPAR-hPWV group was older and more likely had hypertensive baseline characteristic than the other groups, which was confirmed by these studies^[7-9]^. These patients’ PWV values were higher than the reported reference values, in addition to age and hypertension-related increase^[7]^. Also, the best-known effect of increased aortic stiffness is early aortic pulse reflection, which causes an increase in PP due to an increase in systolic blood pressure, and a decrease in diastolic blood pressure. In the present study, patients with high PWV had higher PP, consistent with previous studies^[21]^.

In the present study, 53% of the patients had none, 43% of the clinical outcomes^[13,14]^. Furthermore, the PARTNER trial has found patients had mild, and 4,3% of the patients had more than mild PAR, independent association between mild PAR and late mortality consistent with previous studies^[22-24]^. It is known that residual more after TAVI^[14]^. In addition, the correlation between cardiovascular than mild PAR has unfavourable prognostic affects and increases morbidity and mortality with PWV has been well established the risk of morbidity and mortality after TAVI procedure^[25,26]^. before^[8,9]^. Taken together, degenerative elastic properties of aorta, However, in some studies, the clinical significance of mild PAR which reflects as high PWV, may contribute to clinical deterioration has been evaluated and suggested that it can be a predictor of of the patients with mild PAR, as described in our study.

ARI is a reproducible and quantitative hemodynamic AR degree measurement method used during TAVI procedure, which has been validated before with a high accuracy rate^[13,15]^. We know that impairment of ventricular-vascular coupling by increased aortic stiffness provides additional work to the heart^[5]^. This effect may excessively occur in fragile patients by increased oxygen demand and afterload with a diminished diastolic relaxation of the heart^[6]^. As a result, ARI value is decreased, due to increased left ventricle diastolic pressure and aortic systolic pressure with decreased aortic diastolic pressure. In our study, we found lower ARI value in patients with high PWV, supporting this hypothesis.

NT-proBNP level increase by stretching of the myocardium due to pressure or volume overload, and it is a known strong predictive value of adverse outcomes in patients with cardiovascular diseases^[27]^. Different severity of PAR occurring as a complication after TAVI changes the pressure overload to volume overload and increases the mortality rate^[25,26]^. Accordingly, increased afterload and left ventricular end diastolic pressure due to high PWV may contribute to higher NT-proBNP levels and clinical worsening, as we found higher rate of NYHA class II patients (at least) in this group, supports this hypothesis.

### Limitations

First of all, this is a single-centre study including small number of patients. Secondly, we used TTE instead of TEE during the procedure for PAR grading because all procedures were performed under mild sedation. Finally, patients with mild AR and high PWV had significantly more hypertension than patients with normal PWV and were older as well. These two circumstances might affect the functional status of patients in the first-month follow-up.

## CONCLUSION

In the present study, we demonstrated that impairment of aortic elastic properties, which reflects as high PWV, might contribute to early clinical deterioration especially in patients with mild PAR after transfemoral TAVI procedure. These results are also suggesting that the impact of mild PAR on early functional status may be depending on underlying baseline PWV. However, this is the first study to find this association and need to be supported by larger future trials.

**Table:** 

Authors' roles & responsibilities	
IHK	Substantial contributions to the conception or design of the work; final approval of the version to be published
OS	Substantial contributions to the conception or design of the work; final approval of the version to be published
MK	Analysis of data for the work; final approval of the version to be published
FOS	Interpretation of data for the work; final approval of the version to be published
ODU	Interpretation of data for the work; final approval of the version to be published
SS	Acquisition of data for the work; final approval of the version to be published
SC	Acquisition of data for the work; final approval of the version to be published
SK	Analysis of data for the work; final approval of the version to be published
